# Vitamin E for prevention of oxaliplatin-induced peripheral neuropathy: a pilot randomized clinical trial

**DOI:** 10.1590/S1516-31802013000100006

**Published:** 2013-02-01

**Authors:** Samuel Oliveira de Afonseca, Felipe Melo Cruz, Daniel de Iracema Gomes Cubero, Andrea Thaumaturgo Lera, Fernanda Schindler, Marcia Okawara, Luiz Fernando de Souza, Nataly Pimentel Rodrigues, Auro del Giglio

**Affiliations:** I MD. Master’s degree Student, Discipline of Hematology and Oncology, Faculdade de Medicina do ABC (FMABC), Santo André, São Paulo, Brazil.; II MD, MSc. Researcher and Head of the Residency Program, Discipline of Hematology and Oncology, Faculdade de Medicina do ABC (FMABC), Santo André, São Paulo, Brazil.; III MD, MSc. Adjunct Professor, Discipline of Hematology and Oncology, Faculdade de Medicina do ABC (FMABC), Santo André, São Paulo, Brazil.; IV MD. Resident Physician, Department of Internal Medicine, Faculdade de Medicina do ABC (FMABC), Santo André, São Paulo, Brazil.; V BSc. Pharmacist, Discipline of Hematology and Oncology, Faculdade de Medicina do ABC (FMABC), Santo André, São Paulo, Brazil.; VI Medical Student, Faculdade de Medicina do ABC (FMABC), Santo André, São Paulo, Brazil.; VII MD, PhD. Titular Professor, Discipline of Hematology and Oncology, Faculdade de Medicina do ABC (FMABC), Santo André, São Paulo, Brazil.

**Keywords:** Drug therapy, Vitamin E, Peripheral nervous system diseases, Prevention & control, Colorectal neoplasms, Randomized controlled trials as topic, Quimioterapia, Vitamina E, Doenças do sistema nervoso periférico, /Prevenção & controle, Neoplasias colorretais, Ensaios clínicos controlados aleatórios como assunto

## Abstract

**CONTEXT AND OBJECTIVE:**

Oxaliplatin is one of the chemotherapy regimens most used for treating colorectal cancer. One of the main limitations to its use is induction of peripheral neuropathy. Previous studies have shown that vitamin E can reduce the incidence of peripheral neuropathy by 50%. This study aimed to assess the effectiveness of vitamin E for prevention of oxaliplatin-induced peripheral neuropathy.

**DESIGN AND SETTING:**

Prospective, phase II, randomized pilot study developed at a university hospital in the Greater ABC region.

**METHODS:**

Patients were randomized five days before starting oxaliplatin treatment, to receive either vitamin E or placebo until the end of the chemotherapy regimen. The outcome was evaluated using the Common Terminology Criteria for Adverse Events (CTCAE), version 3, and specific gradation scales for oxaliplatin-induced peripheral neuropathy. Patients with colorectal and gastric cancer who had been scheduled to receive oxaliplatin-based chemotherapy were included. Both groups received calcium and magnesium supplementation before and after oxaliplatin infusions.

**RESULTS:**

Eighteen patients were randomized to the vitamin E group and 16 to the placebo group. Cumulative incidence of 83% with peripheral neuropathy grades 1/2 was observed in the vitamin E group, versus 68% in the placebo group (P = 0.45). A trend towards more diarrhea was observed among patients who received vitamin E (55.6% vs. 18.8%; P = 0.06). There were no other significant differences in toxicity between the groups.

**CONCLUSIONS:**

No significant decrease in the incidence of acute oxaliplatin-induced peripheral neuropathy was demonstrated through vitamin E use.

**CLINICAL TRIAL REGISTRATION:**

NCT01523574

## RESUMO

**CONTEXTO E OBJETIVO:**

A oxaliplatina é um dos quimioterápicos mais utilizados no tratamento do câncer colorretal, sendo a indução da neuropatia periférica (NP) uma das principais limitações para o seu uso. Trabalhos anteriores demonstraram que a vitamina E poderia reduzir a incidência dessa neuropatia em 50%. Este estudo teve como objetivo avaliar a efetividade da vitamina E na prevenção da NP induzida pela oxaliplatina.

**TIPO DE ESTUDO E LOCAL:**

Estudo piloto prospectivo e randomizado de fase II desenvolvido em hospital universitário do Grande ABC.

**MÉTODOS:**

Os pacientes foram randomizados para receber vitamina E ou placebo por cinco dias antes do início do tratamento com oxaliplatina e até o término do regime quimioterápico. O desfecho foi avaliado através dos Critérios Comuns de Toxicidade do Câncer versão 3 (CTCAE) e escalas específicas de gradação da NP induzida por oxaliplatina. Foram incluídos pacientes com câncer colorretal e gástrico programado para receber quimioterapia baseada em oxaliplatina. Ambos os grupos receberam suplementação de cálcio e magnésio antes e depois das infusões de oxaliplatina.

**RESULTADOS:**

Dezoito pacientes foram randomizados para grupo da vitamina E e 16 para o grupo placebo. Observou-se incidência cumulativa de 83% das classes I/II de neuropatia periférica no grupo da vitamina E, contra 68% no grupo placebo (P = 0,45). Observou-se maior tendência à diarreia em pacientes que receberam vitamina E (55,6% versus 18,8%, P = 0,06). Não houve outras diferenças significativas quanto às toxicidades entre os grupos.

**CONCLUSÃO:**

Não foi demonstrada diminuição significativa na incidência de NP aguda induzida por oxaliplatina com o uso de vitamina E.

## INTRODUCTION

Oxaliplatin is a frequently used medication that is part of many chemotherapy regimens for several gastrointestinal malignancies. It is an organoplatinum complex that can produce both inter- and intra-strand platinum-DNA crosslinks that, in turn, lead to inhibition of DNA replication and transcription. One of the most important toxic effects of oxaliplatin is induction of both acute and chronic peripheral neuropathy, which affects most patients treated with oxaliplatin. Peripheral neuropathy can be sufficiently severe to require treatment interruption. The mechanism of oxaliplatin-induced peripheral neuropathy seems to depend on a decrease in extracellular calcium levels, which may impair sodium influx and result in neuronal hyperexcitability.^1^^,^^2^^,^^3^

Vitamin E, which has antioxidant properties, has been evaluated with a view to preventing peripheral neuropathy in patients receiving cisplatin. Pace et al.^4^ evaluated 108 patients who were being treated with cisplatin and randomized them to receive vitamin E at 400 mg/day or placebo. The incidence of neurotoxicity was significantly lower in the group treated with vitamin E (5.9% versus 41.7%; P < 0.01). Furthermore, Argyriou et al. demonstrated a 50% reduction in the incidence of peripheral neuropathy in patients undergoing cisplatin treatment who received 600 mg of oral vitamin E per day, compared with those who received placebo.^5^ Argyriou et al. also confirmed these results in a later study.^6^

Antioxidant medications may play a role in decreasing the peripheral neurotoxicity of platinum-based chemotherapeutic regimens. Cascinu et al. showed that glutathione significantly decreased the rates of peripheral neuropathy induced by oxaliplatin.^7^ These authors demonstrated that, after 12 cycles of oxaliplatin-based chemotherapy, grade 2 to 4 neurotoxicity was observed in 3 out of 21 patients who received glutathione, versus 8 out of 19 who were randomized to receive placebo (P = 0.004).

## OBJECTIVE

To assess the effectiveness of vitamin E for prevention of oxaliplatin-induced acute peripheral neuropathy.

## METHODS

This was a prospective, phase II, randomized study. From October 2009 to November 2010, we included patients at a university hospital located in the Greater ABC region of São Paulo. This study was approved by our Institutional Research Ethics Committee, and patients signed informed consent forms before inclusion in this study.

We included patients with an Eastern Cooperative Oncology Group (ECOG) performance status 0 or 1 who were older than 18 years of age, diagnosed with colorectal or gastric cancer and scheduled to receive oxaliplatin-based regimens (fluouracil, leucovarine and oxaliplatin, FLOX; 5-FU/leucovarine plus axaliplatine, FOLFOX; epirubicin, oxaliplatin and capecitabine EOX; and capecitabine plus oxaliplatin, XELOX). We excluded patients with a previous history of peripheral neuropathy or with symptomatic peripheral neuropathy at entry into the study. We also excluded patients who had received other chemotherapy regimens (except 5-fluorouracil alone) and those currently receiving gabapentin, carbamazepine, amitriptyline, amifostine or multivitamins. Both the study and the placebo group received calcium and magnesium supplements before and after oxaliplatin infusions. This supplementation consisted of calcium gluconate and magnesium sulfate, 1 g each, delivered intravenously over a 30-minute period just before the oxaliplatin infusion and repeated at the same dose after completion of the oxaliplatin infusion. Calcium gluconate and magnesium sulfate were given in the same infusion bag.

Patients were randomized five days before the beginning of the oxaliplatin treatment, to receive either vitamin E at a dose of 400 mg daily or placebo, until after the end of the oxaliplatin-based chemotherapy regimen. Randomization was conducted by means of a sequence of consecutive random numbers that were distributed into consecutive envelopes. These were opened by the pharmacist (F.S.), who accordingly then allocated the patients either to placebo or to vitamin E. All other participants were blinded. Unblinding of the study allocation groups took place only after data-gathering for the study had been concluded, in order to enable analysis of the results.

We analyzed all the data using the intention-to-treat principle. We evaluated the intensity of the peripheral neuropathy using the Common Terminology Criteria for Adverse Events (CTCAE), version 3,^8^ and specific gradation scales for oxaliplatin-induced peripheral neuropathy.^9^

Based on the 50% reduction in peripheral neuropathy associated with vitamin E prophylaxis that was observed in the study by Argyriou et al.,^5^ we designed this study to detect a 50% decrease in the incidence of peripheral neuropathy with a power of 0.8 and a type I error of 0.05.

## RESULTS

From October 2009 to November 2010, 38 patients were recruited into this study. We excluded four patients because of difficulties in contacting them by phone, loss from follow-up soon after entry into the study or missing information that precluded any further analysis. Of the 34 remaining patients, 18 patients were randomized to the vitamin E group and 16 to the placebo group. The patients’ clinical characteristics are presented in Table 1.

**Table 1.** Clinical characteristics of the patients included in this study

Clinical characteristics	Vitamin E	Placebo	P
Age	56 (29-76)	57 (40-71)	
Sex			
Male	10	8	0.70
Female	8	8	
Eastern Cooperative Oncology Group (ECOG)			
0	15	14	0.73
1	3	2	
Type of chemotherapy			
FOLFOX	9	7	0.93
FLOX	5	5	
EOX	4	4	
Primary tumor			
Colon, adjuvant	8	9	0.39
Colon, metastatic	3	0	
Rectal	3	3	
Advanced gastric	4	4	
Radiotherapy	2	3	0.53
Previous chemotherapy	2	2	0.90
Diabetes	2	0	0.16
Alcohol consumption	2	4	0.29

**Figure 1.**
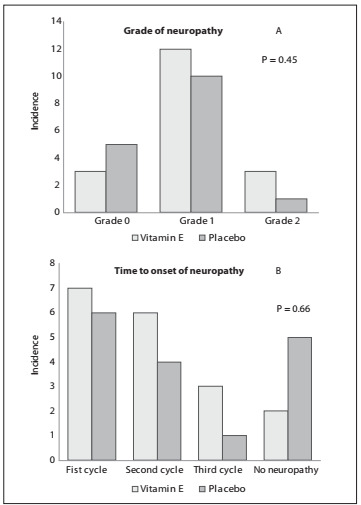
Cumulative number of peripheral neuropathy cases according to intensity, over the course of the chemotherapy treatment period (Graph A). Number of peripheral neuropathy cases according to the cycle of treatment in which they were first identified (Graph B). Grades defined in accordance with the Common Terminology Criteria for Adverse Events (CTCAE) version 3 and a specific gradation scale for oxaliplatin-induced peripheral neuropathy.

Over the course of the study, 83% of the subjects in the vitamin E group developed peripheral neuropathy of grades 1/2, versus 68% in the placebo group (P = 0.45) (Figure 1). We observed a trend towards more diarrhea among patients who received vitamin E (55.6% versus 18.8%; P = 0.06). No other significant differences in toxicity were observed between the groups (Table 2).

**Table 2.** Toxicity observed during the study, according to the Common Terminology Criteria for Adverse Events (CTCAE) version 3 criteria

Grade (CTCAE version 3)	Vitamin E				Placebo				
	1	2	3	4	1	2	3	4	
Diarrhea	5 (14.7%)	5 (14.7%)	0	0	3 (8.8%)	0	1 (2.9%)	0	P = 0.06
Vomiting	6 (17.6%)	2 (5.9%)	0	0	6 (17.6%)	0	0	0	P = 0.38
Nausea	11(32.4%)	4 (11.8%)	0	0	8 (23.5%)	2 (5.9%)	2 (5.9%)	0	P = 0.36
Mucositis	7 (20.6%)	0	0	0	4 (11.8%)	0	0	0	P = 0.38
Fatigue	6 (17.6%)	4 (11.8%)	2 (5.9%)	0	9 (26.5%)	5 (14.7%)	0	0	P = 0.20
Headache	4 (11.8%)	0	0	0	2 (5.9%)	0	0	0	P = 0.45
Vertigo	3 (8.8%)	0	0	0	4 (11.8%)	0	0	0	P = 0.54
Bleeding	1 (2.9%)	0	0	0	1 (2.9%)	0	0	0	P = 0.90

## DISCUSSION

This was a small phase II randomized trial to determine whether vitamin E could prevent oxaliplatin-induced peripheral neuropathy, which can affect up to 80% of treated patients.^1^^,^^10^ Oxaliplatin is a very important medication for treating gastrointestinal malignancies, especially for colorectal, gastric and pancreatic cancers, but its use is often hampered by induction of severe and limiting peripheral neuropathy.^11^^,^^12^

Even though many attempts have been made to prevent this complication, including the use of magnesium and calcium infusions,^13^^,^^14^ oxcarbamazepine^11^ and antioxidants such as glutathione,^7^ peripheral neuropathy remains a challenge in treating these patients.

Vitamin E, which has antioxidant properties, has been studied in relation to prevention of cisplatin-induced peripheral neuropathy, with encouraging results. Therefore, we attempted to evaluate the use of vitamin E in the setting of oxaliplatin treatment.

We could not demonstrate any significant decrease in the incidence of acute oxaliplatin-induced peripheral neuropathy through vitamin E use. The small number of patients we enrolled might have prevented us from finding a smaller difference favoring vitamin E than the difference of 50% that we used in determining the number of patients to include in this trial. Moreover, the vitamin E dose that we used (400 mg) was less than that used by Argyriou (600 mg daily).^5^ However, Pace et al.^4^ reported results similar to those of Argyriou et al.^5^ with a dose of 400 mg daily, which is the same dose as we used in this study. Furthermore, because we used calcium and magnesium supplementation in both groups, we cannot rule out the possibility that an interaction may have occurred between these supplements and the antioxidant activities of vitamin E, which may have rendered vitamin E less effective for preventing peripheral neuropathy in our patients.

Recently, concordant with our findings, a study published by Kottschade et al.^12^ failed to show any significant reduction in the incidence of peripheral neuropathy in patients treated with neurotoxic chemotherapy, even with higher doses of vitamin E (400 mg twice a day). Their study also included 50 patients who received oxaliplatin. These authors observed that the incidence of neuropathy was 34% in vitamin E-treated patients and 29% in those who received placebo (P = 0.43).

The main limitation of the present study was its size. In fact, this was a small randomized pilot clinical trial designed to exclude a 50% or higher difference between groups favoring vitamin E. Even though we cannot recommend vitamin E use on the basis of this small phase II pilot study, further work may be needed in this area in order to evaluate whether vitamin E may still have a role in this setting.

## CONCLUSION

Based on the results from this study, we cannot recommend the use of vitamin E to prevent peripheral neuropathy in patients who are scheduled to receive oxaliplatin.
